# A New Hypothetical Concept in Metabolic Understanding of Cardiac Fibrosis: Glycolysis Combined with TGF-β and KLF5 Signaling

**DOI:** 10.3390/ijms23084302

**Published:** 2022-04-13

**Authors:** Thanachai Methatham, Ryozo Nagai, Kenichi Aizawa

**Affiliations:** 1 Division of Clinical Pharmacology, Department of Pharmacology, Jichi Medical University, Tochigi 329-0498, Japan; d1733@jichi.ac.jp; 2 Jichi Medical University, Tochigi 329-0498, Japan; rnagai@jichi.ac.jp

**Keywords:** cardiac fibroblast, cardiac fibrosis, TGF-β, glycolysis pathway, KLF5

## Abstract

The accumulation of fibrosis in cardiac tissues is one of the leading causes of heart failure. The principal cellular effectors in cardiac fibrosis are activated fibroblasts and myofibroblasts, which serve as the primary source of matrix proteins. TGF-β signaling pathways play a prominent role in cardiac fibrosis. The control of TGF-β by KLF5 in cardiac fibrosis has been demonstrated for modulating cardiovascular remodeling. Since the expression of KLF5 is reduced, the accumulation of fibrosis diminishes. Because the molecular mechanism of fibrosis is still being explored, there are currently few options for effectively reducing or reversing it. Studying metabolic alterations is considered an essential process that supports the explanation of fibrosis in a variety of organs and especially the glycolysis alteration in the heart. However, the interplay among the main factors involved in fibrosis pathogenesis, namely TGF-β, KLF5, and the metabolic process in glycolysis, is still indistinct. In this review, we explain what we know about cardiac fibroblasts and how they could help with heart repair. Moreover, we hypothesize and summarize the knowledge trend on the molecular mechanism of TGF-β, KLF5, the role of the glycolysis pathway in fibrosis, and present the future therapy of cardiac fibrosis. These studies may target therapies that could become important strategies for fibrosis reduction in the future.

## 1. Introduction

A major issue in cardiology medicine is understanding the molecular basis of ventricular remodeling. Changes in the size and function of the heart are found in cardiac remodeling in failing hearts, which is observed from structural rearrangements such as cardiac fibroblast proliferation, fibrosis production, and cardiomyocyte hypertrophy [1]. Due to the limited regeneration ability of the adult mammalian heart after injury, the loss of substantial cardiomyocytes leads to the generation of fibrosis, which is a critical step for maintaining the functional integrity of the myocardial structure [2].

Cardiac fibrosis, a common process in the pathophysiology of most heart diseases, is the outcome of a reparative process for responding to the damage of cardiomyocytes. In general, the processes that cause fibrosis are associated with increased extracellular matrix (ECM) synthesis or reduced ECM degradation. Cardiac fibrosis is characterized by an imbalance in ECM homeostasis by excess ECM deposition produced by cardiac fibroblasts [3,4]. To carry out this role, the cardiac fibroblasts within connective tissue transform into activated myofibroblasts that produce increased quantities of ECM proteins to foster a profibrotic environment [5]. Cardiac fibroblasts play an important function in the maintenance of ECM protein homeostasis [6]. Transitioning the cardiac fibroblasts into myofibroblasts is a crucial stage in the progression of cardiac fibrosis, which leads to heart dysfunction and finally to death. Understanding the process of how fibrosis develops is critical as a means to discover methods to prevent or treat it before it becomes harmful to the heart. 

Many previous studies have found that transforming growth factor-β (TGF-β) plays a key role in heart illnesses such as cardiac hypertrophy and fibrosis, heart remodeling, and heart failure [7]. Importantly, research on Krüppel-like factor 5 (KLF5) has been linked to crosstalk between cardiac fibroblasts and cardiomyocytes [8], and lowering KLF5 expression has reduced fibrosis in the heart in a mouse model with transverse aortic constriction (TAC) [9]. Because cardiac fibrosis is such an important regulatory process in heart failure, a better knowledge of the underlying mechanisms of the fibrotic process has been eagerly sought in the hope of developing specific therapeutics for fibrotic organ damage. Discovering novel diagnostic or therapeutic targets for cardiac fibrosis is critical, since the treatment for fibrosis at this time is still being studied. Recent discoveries in the etiology of fibrosis have resulted in interesting treatment possibilities, notably in the field of metabolic control [10].

Although knowledge of the interlink of TGF-β and KLF5 with metabolism in cardiac fibrosis is limited, we believe that this review of the relationship of TGF-β, KLF5, and metabolism in controlling fibrosis in other organs may aid in understanding the possible pathways for treating fibrosis in the heart. This review may provide the expected hypothesis for guiding the possible ways to study and be more understanding of fibrosis in the heart. Despite the fact that biochemical techniques and molecular mechanisms have been investigated and developed to identify fibrosis in an injured heart, none of the currently available biomarkers meet all of the criteria for effective cardiac fibrosis evaluation in clinical therapy. To point out a possible biomarker for the future, the metabolic control in fibrosis, and the interplay of the main factors involved in fibrosis, this review focuses on discussing the major factors involved in fibrosis pathogenesis, namely TGF-β, KLF5, and the role of the metabolic process in glycolysis. It also discusses therapies and treatment alternatives in the future.

## 2. Cardiac Fibroblast

### 2.1. The Principal Effector Cells in Cardiac Fibrosis: Fibroblasts and Myofibroblasts

The diversity and roles of cardiac fibroblasts in fibrosis have increasingly been identified in recent years. Better understanding of the processes of these cells is a key for controlling collagen production. Cardiac fibroblasts are a type of connective tissue-producing cell and play an essential role in the development of adult hearts by depositing collagen and ECM components in response to a pathological stimulus when the heart becomes activated by injury or inflammation [11]. The origin of cardiac fibroblasts is assumed to be derived from embryonic proepicardium mesenchymal cells [12]. Some studies have found that fibroblasts are produced from resident fibroblast lineages generated during development, while others have reported that cells can transdifferentiate into fibroblasts and play a substantial role in the production of a fibrotic matrix [13]. The ambiguous origin of cardiac fibroblasts needs to be explored more thoroughly for them to be better understood.

Cardiac fibroblasts have received much interest as a contributor to heart function and as a potential therapeutic target. Activated fibroblasts, which are the primary ECM-producing cells, are the fundamental cellular effectors of cardiac fibrosis. Activated fibroblasts may promote cardiomyocyte enlargement directly through paracrine pathways, leading to heart dysfunction [14]. During cardiac injury and progression to heart failure, fibrosis is shown by the activation of cardiac fibroblasts that impact ECM secretion, production, and degradation, including cytokines, chemokines, and growth factors, resulting in the accumulation of collagen fibers and scar tissues [15,16,17,18]. The main fibroblasts are ECM proteins, which secrete types of collagen, including types I and III and fibronectin [19]. The majority of cells in the normal heart are fibroblasts [20]. Myocardial injury or stress causes the fibroblasts to change to myofibroblasts [21,22]. To support the mechanism of the failing heart, myofibroblasts generate and deposit ECM proteins. After resolving the injury, the final step of secreting ECM such as collagen, fibronectin, galectins, and periostin to maintain the damaged tissue is provided by the matrifibrocytes, which are derived from myofibroblasts [21,22]. In myofibroblast activation mechanisms in fibrotic hearts, we now know that the action of myofibroblasts not only causes the secretion of ECM proteins but also secretes enzymes, proteases, and other inhibitors related to matrix remodeling. Chemical mediators, such as growth factors, neuronal and humoral factors, and cytokines, are generated and activated after cardiac damage, causing intracellular fibrosis by attaching to cell surface receptors and transducing signals. Then, in response to damage, inflammatory and fibrogenic programs are activated by generating matrix proteins, immune cells, and macromolecules, which help to functionally activate fibrotic growth factors at the site of injury [23]. The processing of existing ECM to remove damaged tissue, as well as the manufacture, secretion, crosslinking, and growth of new ECM, are all important to understand in comprehending how structured fibrosis develops and in devising a strategy to cure fibrosis in the heart. Furthermore, investigating the molecules and factors that regulate fibroblasts will aid in understanding the mechanism and finding strategies to prevent fibroblasts from transforming into fibrosis.

### 2.2. Current Studies of Molecular Markers for Cardiac Fibroblasts

Due to the phenotypic alterations of fibroblasts in injured tissues, the identification and characterization of fibroblast populations is complicated. There are many studies suggesting the potential utility of markers that are valuable for clinical risk prediction [24,25,26]. Many of the known fibroblast markers, including ECM proteins such as collagen I and collagen III, as well as fibronectin, are highly expressed by fibroblasts [27]. Using a myofibroblast marker such as periostin, several investigations of the origin of myofibroblasts have been conducted, and it has been discovered that local cardiac fibroblasts provide the greatest contribution to the myofibroblast population in cardiac damage [28,29]. Periostin, an ECM secretion for resolving the injured heart, has been proposed as a marker of activated fibroblasts, possibly indicating early fibroblast activation [30]. Smooth muscle α-actin is only expressed during myofibroblast development [27]. Vimentin is prevalent in fibroblasts as well as in myofibroblasts and has been extensively used to label cardiac fibroblasts [31,32]. Moreover, fibroblast-specific protein, discoidin domain receptor 2, thymus cell antigen 1 (Thy1 or CD90), fibronectin, and vimentin are among the cell surface and intracellular markers employed for identification [20,28,33,34].

Several markers have been investigated to label cardiac fibroblasts, as shown in Table 1. The majority of markers are found to be expressed on non-specific cell types or identified as a subset of fibroblasts. Transcription factor 21 (Tcf21), which has been employed as a cardiac fibroblast marker, is present in fibroblasts [35]. Most often utilized is platelet-derived growth factor receptor α (PDGFRα), which is extensively expressed in fibroblasts found in the heart [36]. Although two markers, TCF21 and PDGFRα, are shown to be the most reliable for cardiac fibrosis, neither a single marker nor two markers are sufficiently specific to define cardiac fibroblasts [29,37,38]. To evaluate cardiac fibroblast detection by using these markers, we suggest that the combination of interested markers is appropriate. The identification of fibroblast markers may lead to the discovery of novel candidates for the complex of fibroblast-related cell types observed in a variety of tissues and disease states. Using the variety of fibroblast markers in the early detection and assessment of fibrosis might potentially aid the diagnosis and treatment of heart failure in the early stage and may help physicians to cure fibrosis if it is developing at an early phase of the disease, and to target the therapy for the heart failure patient in the future.

Although research of fibroblast markers has resulted in the development of some fibroblast lineage-tracing tools, only a subset of previously established fibroblast markers is acceptable for fibroblast lineage-tracing, since the majority of those markers are not unique to cardiac fibroblasts. Such tools are also used in cell and mouse models to understand the origin of cardiac fibroblasts and their cell fate changes in response to cardiac injuries [39]. However, the study of molecular mechanisms regulating cardiac fibroblasts, generated fibrosis after injuries, and therapeutic potential is also important. Therefore, we should pay attention to the molecular mechanisms and the involvement of factors in fibrosis.

**Table 1:** List of molecular markers used for the identification of cardiac fibroblasts.

Markers	Biological Character	Biological Function	Expression in Cardiac Fibroblast	Expression in Other Cells	Reference
Thymus cell antigen 1 (Thy1 or CD90)	Cell–cell interaction in cell surface	Membrane glycoprotein for mediating cellular adhesion and communication	Yes	Immune cell, endothelial cell, vascular smooth muscle cells	[34,35,37,40,41]
Collagen I	ECM protein	Targeting collagen I protein-producing cells	Yes	Vascular smooth muscle cells, cardiomyocytes, endothelial cell	[29,42,43,44]
Discoidin domain receptor 2 (DDR2)	ECM-cell interactions mediated via cell surface	Collagen-specific tyrosine kinase receptor mediating cell growth, regulates cell differentiation migration, and proliferation	Yes	Epicardium	[33,45,46,47]
Fibronectin	ECM protein	Migration and growth, cell adhesion, scar healing	Yes	Endothelial cell	[20,48,49]
Fibroblast-specific protein 1 (FSP1)	Calcium binding protein in cytoplasm	Motility, polymerization, cell proliferation, Migration	Yes	Immune cell, endothelial cell, vascular smooth muscle cells	[28,50,51,52,53]
Platelet-derived growth factor receptor α (PDGFR α)	Receptor tyrosine kinases transmit signals from the cell surface into the cell	Tyrosine kinase receptor, proliferation, differentiation	Yes	Cardiac progenitor cell	[29,42,54]
Periostin	ECM protein	Cardiac development, cell migration and adhesion, remodeling, and ECM	No (expressed in cardiac myofibroblast)	Epicardium	[29,30,55,56]
A-Smooth muscle actin (α-SMA)	Cytoskeletal protein	Scar healing, cell migration, and contraction	No (expressed in cardiac myofibroblast)	Vascular smooth muscle cells, epicardium	[28,29,57,58,59]
Transcription factor 21 (TCF21)	Transcription factor in nucleus	Linage specification, regulate mesenchymal cell transitions	Yes	Epicardium	[29,35,60]
Vimentin	Cytoskeleton protein	Intermediate filament protein in cell structure, stabilizing cytoskeletal interactions, cell attachment and migration	Yes	Vascular smooth muscle cells, endothelial cell	[29,61,62,63,64]

## 3. Cardiac Fibrosis

Cardiac fibrosis is defined by increased activity of cardiac fibroblasts, which leads to the accumulation of ECM proteins, causing a higher risk of heart failure [65]. Cardiac fibroblasts evolve in the development of cardiac fibrosis and play an important role in two types of fibrosis, namely reactive cardiac fibrosis (interstitial) and reparative cardiac fibrosis (replacement). Reactive fibrosis occurs without significant cardiomyocyte loss, allowing the heart to adapt to injury and retain its pressure-generating ability [66]. Replacement fibrosis, on the other hand, occurs in response to injury-causing cardiomyocyte death and the replacement of dead cells, resulting in the formation of a collagen-based scar [66]. Various causes of heart failure exhibit different fibrotic processes in the types, characteristics, inductions, and dynamics of fibrosis. The direct molecular processes that underlie the effect of cardiac fibrosis are still being investigated. It is known that the mechanism of cardiac fibrosis has an intricacy of linkages, and a wide spectrum of molecular pathways is implicated in the fibrotic response. 

Various molecules have been identified in the development of cardiac fibrosis, including TGF-β, whose role has been implicated in several studies. The activation of the TGF-β receptor Smad2/3 pathway appears to be the primary mediator of myofibroblast activation and ECM buildup in parts of the signaling of the cell. The study of KLF5 has been linked to cardiac remodeling in the pressure overload model, and the KLF5 fibroblasts are thought to be responsible for the enhanced heart fibrosis caused by pressure overload. Interestingly, inhibiting glycolysis can help to reduce cardiac fibroblast activation and fibrosis after a myocardial infarction (MI) [67,68,69]. In fact, there are many molecules associated with the fibrosis and glycolysis pathway. Since we have found a decrease in KLF5 expression related to TGF-β, as well as an alteration in the metabolic pathway, causing the reduction of fibrosis in a TAC mouse model [9], we focus on the related molecules to explain how they work and how they combine with the glycolysis pathway. Below, we discuss cardiac fibrosis in terms of TGF-β and KLF5 incorporated with metabolic regulation in the glycolysis pathway.

### 3.1. Role of TGF-β

TGF-β, a critical downstream effector of TGF signaling in cardiac fibroblasts, has been identified as an inducer of fibronectin expression and the transcription factor scleraxis, which controls the expression of a broad range of ECM genes and proteins in cardiac fibroblasts [49,70,71,72,73,74]. TGF-β contributes to fibrosis development by encouraging myofibroblast alteration and increasing ECM protein production in active fibroblasts [74,75]. Adhesive interaction linked among activated Th1 cells and cardiac fibroblasts has been hypothesized to initiate fibroblast-derived TGF-β synthesis, which might drive fibroblast to myofibroblast conversion in a pressure-overload model [76]. TGF-β promotes α-smooth muscle actin expression in cardiac fibroblasts, which is a characteristic of myofibroblast conversion [77]. All of these findings show that TGF-β promotes the growth of myofibroblasts in the healing infarction and enhances the production of ECM proteins. As a result, TGF-β is an essential regulator of the fibrosis process.

In a previous study, only three (TGF-β1, TGF-β2, TGF-β3) of five isoforms were found in mammals [71]. Despite the fact that all three isoforms are present in fibrotic tissues, TGF-β1 is the primary cause of tissue fibrosis. TGF-β1 is the most common isoform, present practically everywhere in mammalian tissues, whereas the other isoforms are found in a smaller number of cells and tissues. TGF-β1 levels in the blood and tissue are elevated in human hepatic [78,79], renal [80,81], and pulmonary fibrosis [82], as well as in heart failure [83], suggesting that TGF-β1 is a significant factor in fibrotic diseases in humans. The lack of one TGF-β1 allele appears to reduce age-related cardiac fibrosis and may contribute to increased lifetime survival in mice [84]. In addition, TGF-β1 increased the quantity of mRNA for collagen types I and III in cardiac fibroblasts, causing an increase in collagen synthesis [85]. Three TGF-β isoforms communicate through the same receptors and have identical cellular targets in mammals, but they have distinct regulatory patterns and affinities for their receptors and co-receptors, implying that these isoforms play distinct roles in the pathophysiology of fibrotic diseases [71]. Because of investigations into the three isoforms’ shared downstream signaling pathways, TGF-β’s function in fibrotic diseases is generally acknowledged. The induction of TGF-β has been proven in a number of cardiac fibrosis models, as well as in human hearts with fibrotic cardiomyopathy [86,87,88,89]. TGF-β has emerged as an appealing therapeutic target, due to its critical involvement in the pathophysiology of fibrosis [90]. Several studies point to TGF-β’s function in the development of cardiac fibrosis. TGF-β inhibition reduces fibroblast activation and cardiac fibrosis, while also preventing diastolic dysfunction in pressure-overloaded rats [91]. This suggests that TGF-β has an important role in myocardial fibrosis in pressure-overloaded hearts by activating fibroblasts. 

In a pressure overload model, mice with fibroblast-specific deletion of the type I receptor (TβRI) or the type II receptor (TβRII) showed less cardiac fibrosis, indicating that activation of TGF-β signaling is required for the process of fibrosis [92]. In the canonical pathway, TGF-β1 can induce a Smad-independent signaling cascade [93]. TGF-β has been linked to the pathogenesis of cardiac fibrosis via Smad pathways by binding TGF-β to TβRI on the cell surface, as well as to TβRII, and then connecting to Smads or Smad-independent pathways, with Smad3 signaling being important in the fibrotic remodeling of the infarcted ventricle [3,92]. TGF-β binds to TβRI, triggering phosphorylation of Smad2 and Smad3, which subsequently form a heteromeric complex with Smad4 and translocate into the nucleus to regulate the transcription of target genes, leading to stimulation of the synthesis of ECM protein [15,18,94].

Previous research investigated whether the fibroblast-specific double deletion of Tgfbr1/2 and Smad2/3 protected against TAC-induced cardiac fibrosis [92]. The deletion of Smad2, Smad3, and Smad2/3 had no effect on pressure-overloaded hearts’ decompensation. Although Smad2 deletion had no influence on unfavorable cardiac fibrosis, Smad3 appeared to be essential for the pressure overload-induced fibrotic response [92]. Moreover, Smad3 deletion in activated fibroblasts increased the onset of early systolic dysfunction following pressure overload [95]. Currently, the TGF-β/Smad3 pathway is known to have a role in pressure overload-induced fibrosis. Therefore, we believe that the TGF-β/Smad3 pathway plays an important in cardiac fibrosis, because it stimulates fibroblasts to produce ECM in injured heart tissue. However, further investigation of this pathway may provide clues about how, in the future, to link to other molecules related to cardiac fibrosis, such as KLF5, to cure heart fibrosis.

### 3.2. Crosstalk between the TGF-β and Glycolysis Pathway

Metabolic investigations in pathogenetic analysis have become increasingly prominent in recent years. Metabolic research can aid a better understanding of illness pathogeneses’ roles and mechanisms. Interestingly, TGF-β signaling is implicated in the stimulation of both glycolysis and mitochondrial respiration [96]. The activation of TGF-β-related pathways and an increase in TGF-β expression are both involved in other organs. Lung myofibroblast differentiation and pulmonary fibrosis both need glycolytic reprogramming [97]. Because pulmonary fibrosis is a disorder of glycolytic dysregulation, approaches to inhibit glycolysis have been identified in order to be applied to treating organ fibrotic disorders. To identify metabolic dysregulations associated with organ fibrosis, glycolytic reprogramming by ramping down glycolysis is critical to lung myofibroblast differentiation and pulmonary fibrosis. The stability of hypoxia-inducible factor 1α (HIF-1α), a master regulator of glycolytic enzymes implicated in organ fibrosis, is aided by increased glycolysis in fibroblasts [97]. Increased glycolysis and lactic acid buildup in fibroblasts promote differentiation into myofibroblasts and enhance TGF-β secretion due to metabolic instability [5,97,98,99].

In vitro studies have shown that lactic acid-induced pH acidification activates fibroblasts and enhances TGF-β activity [10]. This finding indicates that the control of TGF-β and glucose metabolism in fibrosis was previously demonstrated, highlighting TGF-β’s relevance as a metabolic regulator. TGF-β1 can also directly activate the glycolytic enzyme 6-phosphofructo-2-kinase/fructose-2, 6-biphosphatase 3 to boost phosphofructokinase-1-mediated glycolysis stimulation [97]. After TGF-β stimulation, lactate dehydrogenase (LDH) is required for myofibroblast development, and TGF-β enhances LDH expression in lung fibroblasts [100,101]. These findings suggest that an increased glycolytic rate is required to sustain myofibroblast activity. Furthermore, in dermal fibroblasts, TGF-β1 increases glycolysis, while inhibiting glycolysis reduces TGF-β1′s pro-fibrotic actions [102]. The stimulation of glycolysis by TGF-β1 is a critical characteristic of the fibrotic phenotype caused by TGF-β1 in skin cells, and enhanced glutaminolysis is also seen in fibroblasts from patients with systemic sclerosis [102]. In renal fibroblasts, TGF-β1 induces a shift in metabolism from oxidative respiration to aerobic glycolysis [103]. TGF-β1-induced metabolic changes in fibroblasts show that downstream changes in H3 acetylation may be part of a much wider interconnected metabolic and acetylation reprogramming in fibrosis [103]. 

The interplay between TGF-β and the glycolysis pathway in other organs suggests that TGF-β is a significant regulator of glycolysis in fibrosis activity. However, in the heart, much less is known about the role of TGF-β in glycolysis. The intracellular renin-angiotensin system was activated when rat cardiac fibroblasts were exposed to high glucose levels in vitro, resulting in enhanced TGF-β and collagen I production by cardiac fibroblasts [104]. High glucose has been demonstrated to enhance collagen production and TGF-β1 expression in cardiac fibroblasts, but the mechanism is still unknown [105,106]. The regulation of glucose may affect the expression of TGF-β and the production of collagen that contributes to fibrosis. Studying how TGF-β works in the glycolysis pathway during the fibrosis process in the heart is imperative, and may eventually provide clues and techniques for preventing fibrosis.

Although information on TGF-β and glycolysis is still not particularly specific to fibrosis in the heart, this review considers fibrosis in other organs in the hope that this will provide a predicted hypothesis for directing various strategies to investigate and understand heart fibrosis. In the future, the involvement of TGF-β in metabolic pathways, such as the glycolysis pathway, may be critical in unraveling the causes of cardiac fibrosis, becoming an important strategy for fibrosis reduction and healing in clinical therapeutics.

### 3.3. Role of KLF5

Fibroblast expansion and activation after cardiac damage is crucial for healing but may potentially lead to fibrosis, remodeling, and dysfunction. Transcription factors have been reported to control a cardiac fibroblast, and thus to play a crucial role in transcriptional regulation during cardiac hypertrophy. Recently, transcription factor KLF5, a member of the Krüppel-like factors family, has been studied for its involvement in cardiac function. KLF5 controls fibrosis in the heart, kidneys, liver, skin, lungs, and other organs [107]. During pressure overload, fibroblast KLF5 is also responsible for increased cardiac fibrosis [108,109]. The expression of KLF5 has been shown to decrease in TAC mice treated with ICG001, a specific inhibitor of the Wnt/β-catenin signaling pathway that inhibits β-catenin/cyclic AMP response element-binding (CREB) protein transcription, resulting in the reduction of fibrosis [9]. KLF5 has been linked in certain studies as a pro-fibrotic protein [110]. KLF5 is responsible for controlling the release of IGF-1, which is a mediator produced by fibroblasts appearing to regulate the connection between cardiomyocytes and cardiac fibroblasts [8]. *KLF5* haploinsufficiency prevents moderate-intensity pressure overload-induced cardiac fibrosis and hypertrophy [8]. *KLF5* deletion in cardiac fibroblasts has improved cardiac hypertrophy and fibrosis, demonstrating that KLF5 in fibroblasts is crucial for pressure-overload response [8]. Knockout of KLF5 in fibroblasts reduces fibrosis, but knockout of KLF5 in cardiomyocytes causes fibrosis. The association of different cells is important, because knockout in myocardial fibroblasts is prone to heart failure at the cost of reduced fibrosis. 

These findings show that KLF5 is critical in modulating cardiac remodeling and is related to the alteration of fibrosis. Despite the fact that KLF5 is vital in pressure overload-induced cardiac hypertrophy, the specific involvement of KLF5 in cardiac hypertrophy and heart failure is still unclear. Further research on KLF5 is still needed to provide a precise novel strategy for preventing and treating heart failure. KLF5 may be a key regulator for responding to fibrosis, and further research into its signaling will aid our understanding of cardiac fibrosis and pathogenic tissue fibrosis. KLF5 may be potentially developed for detection in the future as a new marker for cardiac fibroblast and/or fibrosis.

In addition, KLF5 is activated and proliferates smooth muscle cells and fibroblasts in vascular lesions in response to cardiac tissue injury, according to experiments in vascular tissues [111]. KLF5 and its genetically downstream target gene, the platelet-derived growth factor-A (PDGF-A) chain, are important regulators of stress-induced cardiovascular remodeling [112]. In response to pathological stimuli and their associated pathologies in the cardiovascular system, KLF5 mediates a delayed persistent induction of the PDGF-A chain in response to phorbol ester [112]. In response to Ang II, KLF5 controls the PDGF-A transcription in vivo, a well-known growth factor involved in mesenchymal-cell activation, angiogenesis, and tissue remodeling [113]. The activation of mesenchymal cells, angiogenesis, and the creation of ECM all have a role in the pathogenesis of atherosclerosis and heart failure. KLF5 is also expressed in myocardial myofibroblasts, and Ang II loading causes considerably less cardiac fibrosis and hypertrophy in *KLF5*^+/−^ mice [114]. This indicates that the knockout of KLF5 can minimize the cardiac hypertrophy and fibrosis caused by Ang II infusion. Ang II activates TGF-β expression via inducing KLF5 expression. Due to the lower expression of TGF-β in hearts of *Klf5*^+/−^ mice, it can also protect *KLF5*^+/−^ mice from cardiac hypertrophy and fibrosis [114]. Modulating TGF-β expression by KLF5 has been shown to reduce fibrosis development in *Klf5*^+/−^ mice; hence, KLF5 may be important for cardiac fibrosis through controlling TGF-β. 

Both molecules will have an influence on fibrosis if they are controlled. Because KLF5 induces ECM reorganization and can activate the promoter of PAI-1, which is an inhibitor of fibrin degradation, it contributes to accumulating ECM and leads to vascular wall thickening and cardiac hypertrophy [115]. In addition, TGF-β, which is downstream of KLF5, regulates the synthesis of several ECM components, including collagen, elastin, and fibronectin, all of which are critical in ECM deposition and fibrosis [114,116]. Disturbed TGF-β signaling can cause atherosclerosis and cardiac fibrosis [117]. In cell culture studies, TGF-β plays a key role in endothelial and SMC proliferation, differentiation, sprouting, tube formation, and migration [117]. TGF-β may induce the endothelial-to-mesenchymal transition, which has been linked to the creation of heart valve cushions and a variety of pathological vascular processes [117]. These findings may support the interplay between the KLF5 and TGF-β in the cardiac vascularization and fibrosis process. Unfortunately, the study of KLF5 and TGF-β in the heart is still limited. Understanding the role of these molecules in fibrosis will help researchers have a better understanding of how to regulate the disease in the future.

Moreover, KLF5 is also related to the fibrotic response in other tissues and organs. In the intestine, KLF5 is a highly active protein in the intestinal epithelium [118]. Mitogens activate KLF5, and its overexpression in fibroblasts causes enhanced proliferation and an altered phenotype [118]. KLF5 has been linked to the development of renal fibrosis through stimulation of the HIF-1α-KLF5-TGF-β1 pathway [119], and silencing of the protein has relieved renal fibrosis produced by high-dose MK-treated HK-2 cells by lowering TGF-β and fibronectin expression [119]. *KLF5* haploinsufficiency in the mouse kidney resulted in decreased accumulation of CD11b^+^F4/80^lo^ cells, which expressed proinflammatory cytokines and induced apoptosis among renal epithelial cells, as well as increased CD11b^+^F4/80^hi^ macrophages that expressed CD206 and CD301 and contributed to fibrosis, in part through TGF-β-production [120]. In the liver, KLF5 is involved in the fibrotic response, as shown by the elevation of KLF5 mRNA expression induced by dimethylnitrosamine, causing liver fibrosis [121]. One of three hallmarks of systemic sclerosis discovered in mice with double heterozygous *Klf5* and *Fli1* deficiency is fibrosis and vasculopathy of the skin and lung [107]. This demonstrates that KLF5 is the most important element in fibrosis modulation. Although the role of KLF5 in other organs may not be the same as its role in the heart, there are some mechanisms that assist and guide the research strategy for the heart.

In addition, other KLFs are also involved in the fibrosis process. KLF4, KLF5, KLF6, and KLF15 have received attention in fibrosis research. In particular, KLF6 is prominent in cardiac fibrosis. KLF6 is involved in activating the TGF-β signaling pathway. Upregulation of KLF6 following acute stress, such as myocardial infarction and Ang II, subsequently induces downstream TGF-β, resulting in pathological cardiac fibrosis [122]. Knockdown of KLF6 in cardiac myocytes in mice reduces cardiac fibrosis after Ang II, suggesting a role for KLF6 in propagating fibrosis, but knockdown of KLF6 in cardiac myofibroblasts does not affect fibrosis [123]. These findings point to a pivotal role for KLF6 in cardiac fibrosis, although in different ways when compared with KLF5. However, the involvement of KLF6 in glycolysis pathways in the heart remains unknown. Therefore, we will mainly discuss KLF5.

### 3.4. Crosstalk between the KLF5 and Glycolysis Pathway

Since TAC mice treated with ICG001 show a reduction in KLF5 expression and metabolic alteration in the heart [9], we focused on the change in glycolysis in relation to KLF5. Unfortunately, little is currently known about the interplay between KLF5 and glycolysis in the heart. However, we hypothesized that the role of KLF5 in glycolysis is also important. 

Here, we mention the key role that KLF5 plays in cancer; although its role in cancer may differ from that in the heart, some parts of its role may help to link knowledge of both for further investigation in the future. Due to HIF-1α being a master regulator of glycolytic enzymes implicated in organ fibrosis, we think that HIF-1α may be a mediator in playing a role among TGF-β, KLF5, and the glycolysis pathway. Previous works have shown KLF5 to be hypoxia-regulated in tumors, and vice-versa, and to work as an upstream regulator of HIF-1α in hypoxic pulmonary hypertension [124,125,126]. In fibrosis, HIF-1α causes excessive ECM, vascular remodeling, and ineffective angiogenesis, all of which exacerbate chronic hypoxia and worsen pathofibrogenesis [127]. HIF-1α-induced glycolysis is thought to be important in generating chemoresistance in lung cancer cells [128]. According to a previous study, knocking down KLF5 in lung cancer cells reduces hypoxia-induced cell survival and accelerates cell death [126]. As a result, blocking the upstream of HIF-1α-induced glycolysis may be a promising strategy for reversing hypoxia-induced chemoresistance. During hypoxia, metabolic adjustments are made to sustain cellular function and stability. The transcriptional pathways involved in the metabolism of the heart under hypoxia are still poorly understood. In the heart, endothelial HIF-1α knockdown has boosted TGF-β signaling, resulting in pathological remodeling [129].

According to a study of the TAC mouse model, cardiac-specific HIF-1α overexpression exacerbates heart failure [130]. However, another study revealed that HIF-1α and its role in angiogenesis during cardiac hypertrophy is essential for an adaptive mechanism [131]. These findings suggest that HIF-1α’s activation capabilities and responsibilities may vary by cell type inside the organ. Therefore, the control of KLF5 to HIF-1α may show a response to varied HIF-1α activation capabilities and responsibilities in the heart and a response to the metabolic alteration. Since the metabolic alteration by shifting fatty acids to glucose is linked to studies of patients and mouse models of diabetes or ischemic cardiomyopathy showing an increase in the expression of cardiac KLF5 [132,133,134,135], the relation between KLF5 and metabolic alteration in glycolysis in the heart should be considered. A better understanding of the role of KLF5-regulated pathways in pathological remodeling will accelerate the development of novel therapeutic strategies. However, we should consider that the remodeling of the heart in each model, such as pressure overload and ischemic remodeling, will be different in their processes and in each location of the heart. KLF5 may potentially be developed for detection in the future as a new marker for cardiac fibroblast and/or fibrosis that is linked to the metabolic pathway.

Although nothing is known about the KLF5 and glycolysis in the heart, we propose that the interconnected knowledge of the molecular mechanism and metabolism will help to reveal the possible pathways for further studying fibrosis in the heart. Indeed, the present understanding of KLF5 and glycolysis via related TGF-β and HIF-1α in fibrosis is only the expected hypothesis for investigating cardiac fibrosis in the future; we point out that the function of KLF5 and related factors in cancer may aid and guide the possible ways to study and provide more understanding of fibrosis in the heart. In the future, this might be a revolutionary strategy to investigate cardiac fibroblasts and fibrosis, combining research on several factors related to fibroblasts and fibrosis in metabolism.

### 3.5. Glycolysis Pathway

In recent years, metabolic research has grown in importance in pathogenetic analysis. The study of glycolysis can help us better understand the role and mechanisms of disease pathogenesis. Pyruvate, the final product of glycolysis, is either transformed to lactic acid (anaerobic glycolysis) or to acetyl-CoA for use in the citric acid cycle (aerobic glycolysis) by mitochondria. Aerobic glycolysis is thought to be involved in a variety of fibrotic disorders, including radiation-induced skin fibrosis, renal fibrosis, pulmonary fibrosis, and liver cirrhosis [97,136,137,138]. When the glycolysis route is suppressed by blocking the glycolytic enzymes hexokinase and pyruvate kinase, the pro-fibrotic reaction is diminished [97,136,139]. By inhibiting glucose transporters, it can also reduce pro-fibrotic effects [136]. In cancer cells, the increase in glycolytic intermediates is hypothesized to feed subsets of pathways that promote the actions of the proliferating cells via the Warburg effect [140]. A reverse Warburg effect has been proposed in fibrosis, contributing to pathogenesis, in which fibroblasts participate in aerobic glycolysis and glycolytic metabolites, impacting the activity of other cell types, such as epithelial cells and macrophages [141]. 

Patients with diabetes mellitus have fibrosis in the organs, such as the kidneys, heart, liver, and eyes, and peritoneal dialysis patients are at risk of peritoneal fibrosis due to chronic high-glucose dialysate [142,143]. In diabetic kidneys, SIRT3 deficiency increases aberrant glycolysis, which is responsible for the fibrogenic pathway, and inhibiting abnormal glycolysis by SIRT3 restoration can prevent diabetes-related kidney fibrosis [67]. In the heart, cardiac fibrosis occurs in conjunction with an increase in cardiac fibroblast activation and glycolysis [144]. After an MI, inhibiting glycolysis can help to reduce cardiac fibroblast activation and fibrosis [67,68,69]. Glycolysis may be induced in the canine atrium by paroxysmal atrial fibrillation, and inhibiting glycolysis can entirely reverse myocardial fibrosis remodeling [145]. 

It is possible that increased glycolysis may have more negative consequences on the heart than positive effects. Glycolysis is elevated in cardiac hypertrophy, which can lead to heart failure [146,147]. The function of the Warburg effect in cardiac disease is now being researched [148]. Interfering with the Warburg effect improves cardiac function in heart failure [148]. The failing heart may experience a parallel Warburg effect via an increase in glycolysis and a loss in mitochondrial oxidative metabolism, as the Warburg effect indicates the induction of an increase in the rate of glucose absorption and the formation of lactate [148,149,150,151]. However, no direct study has been conducted to support this notion. Although the mechanism of the Warburg effect in the heart is still unclear, the findings from these studies may help to identify consequences for use in future studies. 

Furthermore, in tissue from humans with heart failure, the expression of the fetal M2 isoform (PKM2), which is a hallmark of the Warburg effect, increases significantly [151,152]. The transformation from the adult M1 isoform of pyruvate kinase (PKM1) to PKM2 is promoted by the activation of HIF-1α [152]. HIF-1α, a master regulator of glycolytic enzymes implicated in organ fibrosis, is thought to play a role in its regulation [148]. During acute ischemia in adult murine cardiomyocytes, glycolytic capacity is increased by HIF-1α [153], which correlates with previous work mentioning that induction of glycolytic pathway enzymes is caused by the HIF-1α-driven response to hypoxia [154]. Next, PKM2 induction is an indication of sunitinib cardiotoxicity, as well as a stressed heart in general [152]. In the hearts of mice, sunitinib increases glucose absorption and causes a metabolic switch to aerobic glycolysis [152]. These findings imply that inhibiting the glycolysis process directly, or molecules involved in the activation of the glycolysis pathway indirectly, may help to prevent fibrosis. As a result, TGF-β and KLF5 modulation via HIF-1α may alter glycolysis control in the fibrosis process. An exact understanding of the metabolic abnormalities may lead to the discovery of novel therapeutic targets.

## 4. Therapies and Treatment Alternatives in the Future Direction

Fibrosis, a key factor in heart failure and its development, is a process seen in cardiac remodeling. The response to fibrosis by fibroblast activation and proliferation is essential for tissue repair. To establish novel treatment targets or methods, it is vital to understand the specifics of the condition, which contributes to pathological and physiological tissue remodeling. Understanding how the injured heart has been developed by modifying scar characteristics to improve the wound may be the key to attaining a novel approach to cardiac therapy [155]. Studying the relation of function, phenotype, and pharmacological activities of fibroblasts in cardiac activity is critical for developing treatment options for wounded hearts. 

The use of cardiac fibroblast marker tracing to follow fibroblast alterations might be an initial step in tracking the fibrosis process. Next, angiotensin-converting enzyme inhibitors or mineralocorticoid receptor antagonists, ATI receptor antagonists, β-blockers, endothelin antagonists, statins, and pirfenidone (a specialized anti-fibrotic medication) have all been used as pharmacological therapies for treating fibrosis in heart failure [156,157,158]. The clinical relevance of these findings remains unknown. 

Although there are now medications available to treat fibrosis, the effects of drug therapy have received little consideration. The path to therapy must be recognized as a significant component in the development of failing heart treatment, to control fibrosis for the prevention of heart failure. Because existing guidelines and classifications of heart failure patients do not consider several different pathological mechanisms for underpinning fibrosis production, fibrosis is not yet the main treatment target for heart failure. Fibrosis-specific mediators in pathways such as TGF-β contribute to the progression of fibrosis, and the inhibition of the TGF-β factor is found in preclinical and clinical trials. Tranilast and pirfenidone are two promising antifibrotic drugs in preclinical and clinical trials for treating cardiac fibrosis by inhibiting the effects of TGF-β and other pathologic growth factors [158]. TGF-β involved in fibrosis development has been found to be controlled by KLF5 [114]. Disease-specific biomarkers will aid in the identification of individuals who may benefit from a specific medication in clinical treatment. 

**Figure 1:**
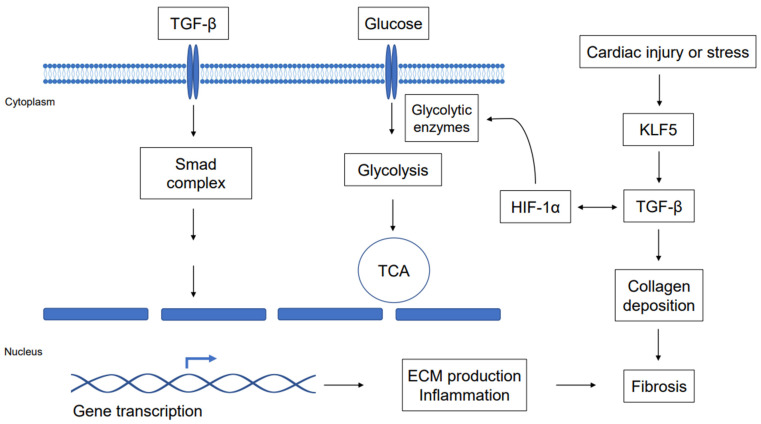
Cooperation of TGF-β-Smad signaling fibrosis, the glycolysis pathway, and KLF5 as regulators of fibrosis. The stress or injury in the heart upregulates KLF5 and subsequently induces TGF-β. In TGF-β-Smad signaling, TGF-β activates the Smad complex, resulting in an increase in the production of ECM and causing fibrosis. Moreover, HIF-1α may regulate TGF-β in fibrosis development and control the glycolytic enzymes in the glycolysis pathway.

This review suggests that KLF5 should also be a promising candidate for a prognostic biomarker and therapeutic target in cardiac fibrosis and heart disease. Moreover, glycolysis may be a novel therapeutic target for heart fibrosis [144]. The connection between TGF-β, KLF5, and the glycolysis pathway may help to identify individuals who may benefit from a particular treatment of fibrosis in clinical therapy. We suggest cooperation among the TGF-β-Smad signaling fibrosis, the glycolysis pathway, and KLF5 as regulators of fibrosis, to provide alternative pathways for studying the treatment of cardiac fibrosis in the future (Figure 1). Eventually, the use of systems biology techniques, biomarkers, and metabolic patterns will assist in the network’s knowledge of fibrosis prevention and therapy. Further research into the relationship between fibrosis pathogenesis, key regulatory factors that affect cardiac fibrosis, changes in the metabolism associated with fibrosis in the heart, and biomarkers for elucidating fibrosis pathways should be conducted for developing effective new methods to prevent the occurrence and development of cardiac fibrosis. To develop successful therapeutics for fibrotic disease, it is necessary to consider the inflammation that causes fibrosis, as well as to conduct extensive research on the specific molecules and metabolic pathways that are challenging for treatment targets and pathways of the inflammatory response to fibrosis in the heart.

## 5. Conclusions

In conclusion, we have summarized the current knowledge of cardiac fibroblast and fibrosis, and we have provided the relationship of the factors for fibrosis among TGF-β, KLF5, and glycolysis pathways. The knowledge gaps in the factors for fibrosis can be considered to be a new therapeutic target for cardiac fibrosis. Although there are many different factors and forms of cardiac fibrosis, the processes that promote the growth of fibrosis and the formation of heart scars are still poorly understood. The identification of the factors and the relative contribution of fibrosis to cardiac dysfunction has been raised. The review of TGF-β, KLF5, and the interconnected metabolism for fibrosis appears to provide the expected hypothesis for leading potential strategies to investigate and comprehend fibrosis in the heart. The application of systems biology approaches, biomarkers, and metabolic patterns may aid in the network’s understanding of cardiac fibrosis and how we monitor and treat it before it progresses to the severity of heart failure. Altogether, the available and emerging molecular and metabolic information, and continued clinical trials, will present challenges and provide hope that new treatment options will become available for fibrosis treatment in the future.

## Data Availability

Not applicable.
